# Clinical Outcomes of Primary Versus Revision Hip Arthroscopic Surgery: A Systematic Review and Meta-analysis

**DOI:** 10.1177/03635465251324944

**Published:** 2025-03-30

**Authors:** Muzammil Akhtar, Daniel Razick, Mustafa Jundi, Jamal Zahir, Sonia Aamer, Anand Dhaliwal, Trevor Shelton, Dean Wang

**Affiliations:** * California Northstate University College of Medicine, Elk Grove, California, USA; † University of California, Davis School of Medicine, Sacramento, California, USA; ‡ Department of Orthopedic Surgery, Valley Consortium for Medical Education, Modesto, California, USA; § Intermountain Health, Provo, Utah, USA; ‖ Department of Orthopaedic Surgery, University of California, Irvine, Irvine, California, USA; Investigation performed at the University of California, Irvine, Irvine, California, USA

**Keywords:** hip arthroscopic surgery, primary, revision, patient-reported outcomes

## Abstract

**Background:**

As the incidence of primary hip arthroscopic surgery has increased, the incidence of revision hip arthroscopic surgery has also increased. Although many factors have been reported that predict clinical failure of hip arthroscopic surgery, the outcomes of primary versus revision hip arthroscopic surgery are unknown.

**Purpose:**

To perform a systematic review and meta-analysis comparing the outcomes of primary versus revision hip arthroscopic surgery.

**Study Design:**

Systematic review and meta-analysis; Level of evidence, 3.

**Methods:**

A search following PRISMA (Preferred Reporting Items for Systematic Reviews and Meta-Analyses) guidelines was performed in the PubMed, Embase, and Cochrane Library databases. Studies were included if they compared the outcomes of primary versus revision hip arthroscopic surgery and had a minimum follow-up of 12 months. Data regarding study characteristics, patient characteristics, radiographic parameters, patient-reported outcomes, and adverse events were recorded. A meta-analysis was conducted using a random-effects model.

**Results:**

There were 11 studies included, with 6437 patients (56.1% female; mean age, 37.1 years) and 1151 patients (65.3% female; mean age, 35.2 years) undergoing primary and revision hip arthroscopic surgery, respectively. Preoperative and postoperative radiographic parameters were not clinically different between the primary and revision groups. Postoperative scores for the Hip Outcome Score–Activities of Daily Living, Hip Outcome Score–Sports-Specific Subscale, modified Harris Hip Score, International Hip Outcome Tool–12, and Non-Arthritic Hip Score were significantly lower (all *P* < .001), and the visual analog scale for pain (*P* < .001) score was significantly higher, after revision hip arthroscopic surgery. For the primary versus revision group, the rate of achieving the minimal clinically important difference ranged from 66.7% to 92% versus 47.4% to 90%, respectively, and the rate of achieving the Patient Acceptable Symptom State ranged from 52.6% to 79.4% versus 20% to 64%, respectively. The risk of complications (*P* = .04) and conversion to total hip arthroplasty (*P* < .001) was significantly higher after revision hip arthroscopic surgery.

**Conclusion:**

Patients undergoing revision hip arthroscopic surgery were less likely to achieve clinically significant improvements in postoperative patient-reported outcomes and exhibited a higher risk of complications and conversion to total hip arthroplasty compared with patients undergoing primary hip arthroscopic surgery. These findings suggest that outcomes are optimized in the primary setting, and surgeons should appropriately counsel patients regarding expectations after revision hip arthroscopic surgery.

Hip arthroscopic surgery is a minimally invasive procedure utilized for the diagnosis and management of hip joint abnormalities such as femoroacetabular impingement (FAI), labral tears, and chondral injuries.^37^ The popularity of hip arthroscopic surgery has significantly increased in the past few decades because of its minimally invasive nature, allowing for lower complication rates compared with open surgery, and favorable outcomes.^10,32^ Many studies have reported on trends in hip arthroscopic surgery performed by orthopaedic surgeons, with the American Board of Orthopaedic Surgery database reporting an 18-fold increase in hip arthroscopic procedures performed between 1999 and 2009.^7^ Similarly, another study reported a 495% increase in hip arthroscopic procedures from 2004 to 2016 in the state of New York, with the largest increase being in the 10- to 19-year age group.^35^

While the incidence of hip arthroscopic surgery has increased, so too have the rates of revision hip arthroscopic surgery.^14^ While reasons for the failure of hip arthroscopic surgery may be highly variable, reported factors associated with an increased risk of revision surgery include age >50 years, diagnosis of osteoarthritis, residual FAI morphology and acetabular dysplasia, iatrogenic chondrolabral injury, joint space <2 mm, female sex, increased acetabular coverage, and increased offset in the superior portion of the femoral neck.^3,8,13,30,33,39^ A higher surgeon volume of hip arthroscopic procedures and labral repair performed in the primary setting were associated with a lower rate of revision.^8^

A systematic review in 2020 found that while patient-reported outcomes (PROs) improved significantly from baseline to follow-up after revision hip arthroscopic surgery, the mean PROs were lower for revision cases compared with primary hip arthroscopic surgery.^28^ The most common indication for revision surgery was reported to be inadequate bony resection during the primary procedure. Furthermore, the rates of conversion to total hip arthroplasty (THA) and repeat revision hip arthroscopic surgery ranged from 0% to 14.3% and 2% to 14%, respectively. However, that study did not perform a comparative analysis of the outcomes between primary and revision hip arthroscopic surgery.^28^

The present systematic review and meta-analysis aimed to compare the outcomes of primary versus revision hip arthroscopic surgery in terms of PROs, achievement rates of clinically significant outcomes, surgical complications, and the need for further surgical interventions. We hypothesized that patients undergoing primary hip arthroscopic surgery would demonstrate significantly better outcomes compared with those undergoing revision hip arthroscopic surgery.

## Methods

### Search Strategy

A search following PRISMA (Preferred Reporting Items for Systematic Reviews and Meta-Analyses) guidelines was performed on June 2, 2024, in 3 databases: PubMed, Embase, and Cochrane Library. The following search terms were used to perform the systematic review: (primary AND revision AND hip AND arthroscop*). Studies were included if (1) they compared the outcomes of primary versus revision hip arthroscopic surgery, (2) the mean age of patients was at least 18 years, and (3) patients had a minimum follow-up of 12 months. The exclusion criteria included case reports, review articles, cadaveric studies, technique articles, studies performed on animals, articles not in English, expert opinions, studies on pediatric patients, and studies in which patients had a follow-up <12 months.

### Study Selection

Overall, 2 independent reviewers evaluated studies from the initial database search according to the eligibility criteria. If they were not unanimous in their decision, a third reviewer was consulted to determine study inclusion or exclusion. All included articles underwent a rigorous search of their reference lists to determine whether additional studies fit the inclusion criteria and could be added to the systematic review.

### Data Extraction

Variables extracted from the studies included author, level of evidence (LOE), journal, study design, publication year, study period, patient inclusion and exclusion criteria, number of patients, patient age, follow-up duration, indications for hip arthroscopic surgery, arthroscopic procedures performed, intraoperative findings, preoperative and postoperative radiographic parameters, preoperative and postoperative PROs, achievement rates of clinically significant outcomes, surgical complications, and rates of revision and conversion to THA. All extracted data were compiled for analysis using Word (Office 2011; Microsoft).

### Methodological Quality Assessment

Methodological quality was assessed using the Methodological Index for Non-Randomized Studies (MINORS) score. There were 2 authors (M.A. and D.R.) who rated each article in the systematic review. Each author rated the article individually before the authors reviewed their scores, and any discrepancies were resolved by re-evaluating the articles until a unanimous consensus was obtained.

### Statistical Analysis

Descriptive statistics such as means, percentages, standard deviations, ranges, medians, and interquartile ranges are reported in this review when applicable and when provided by the individual studies. *P* values are also reported when available to show whether a given variable was significantly different between the primary and revision groups. *P* < .05 was considered statistically significant. The mean difference (MD), 95% confidence interval (CI), and *P* value of postoperative radiographic parameters and PROs were calculated. The risk ratio (RR), 95% CI, and *P* value of complications, revision, and conversion to THA were also calculated. The meta-analysis was performed using a random-effects model. Heterogeneity was determined using the *I*^2^ statistic. Forest plots were generated using Review Manager (Version 5.4.1; Cochrane). Weighted means and standard deviations of postoperative PROs were calculated using R (Version 4.3.0).

When calculating the RR, if the events of complications, revision, or conversion to THA were 0 in both the primary and revision groups for a single study, this would result in a division by 0 errors, thus not allowing for calculation of the RR. If that were the case for any study in the meta-analysis, then the Haldane-Anscombe correction was performed in which 1 event was added and 1 patient was added to the total number of patients in each study, allowing for approximation of the RR with strong accuracy.

## Results

**Figure 1.**
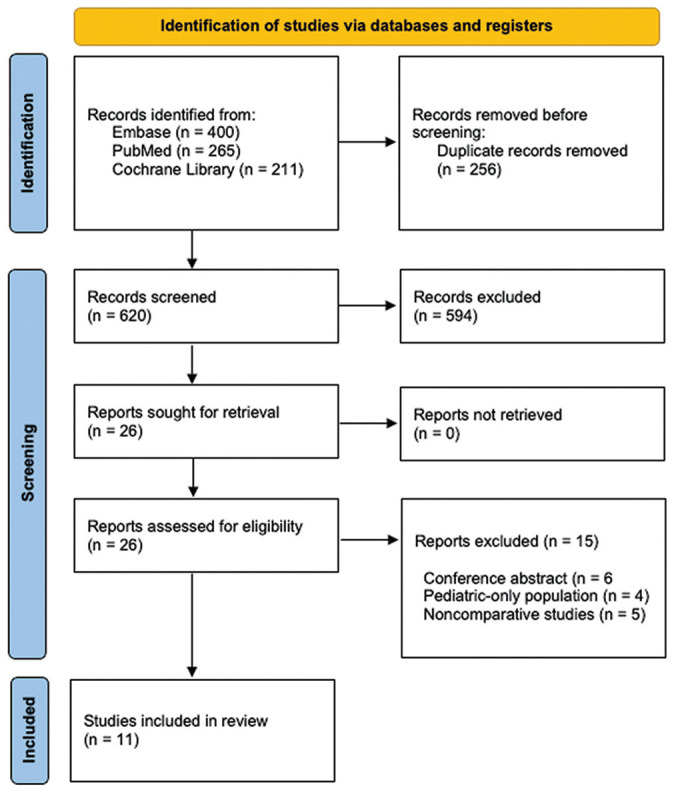
Flow chart depicting the article selection process.

After an initial search of the databases, 876 studies were identified, of which 256 duplicates were removed. The remaining 620 studies underwent title and abstract screening, after which 594 were deemed irrelevant based on predetermined inclusion and exclusion criteria. The remaining 26 studies underwent a full-text review, of which 15 were excluded based on the inclusion and exclusion criteria. As such, 11 studies were chosen to be included in this systematic review.^4-6,9,17-19,25,26,38,40^ The PRISMA flow diagram depicting our search strategy and method of selecting articles is provided in Figure 1.

### Study and Patient Characteristics

Of the included studies, 8 had a retrospective design, and 3 had a prospective design. Also, 2 studies had an LOE of 2, and 9 studies had an LOE of 3. The MINORS score in the studies ranged from 18 to 22. The studies were published between 2014 and 2024, with the study period ranging from 2005 to 2021. There were 6437 patients (43.9% male, 56.1% female) and 1151 patients (34.7% male, 65.3% female) who underwent primary and revision hip arthroscopic surgery, respectively. The pooled mean age was 37.1 years (range, 29.2-50.3 years) and 35.2 years (range, 29.1-49.5 years) for the primary and revision groups, respectively. The follow-up time for the primary and revision groups ranged from 12 to 74.4 months and 12 to 76.8 months, respectively (Table 1). The indications for primary hip arthroscopic surgery predominantly included FAI, labral tears, and borderline hip dysplasia. The indications for revision hip arthroscopic surgery predominantly included residual FAI, labral retears, and capsular insufficiency (see Appendix Table A1, available in the online version of this article). In the majority of studies, the incidence of labral repair, acetabuloplasty, and femoroplasty was significantly higher in the primary group, whereas the incidence of labral debridement and reconstruction was significantly higher in the revision group (see Appendix Table A2, available online). Preoperative osteoarthritis based on the Tönnis grade and intraoperative labrum and cartilage findings are presented in Table 2.

**Table 1:** Study and Patient Characteristics^*a*^

Author	LOE	Study Design	MINORS Score	No. of Patients (M/F)		Age, y		Follow-up, mo	
				Primary	Revision	Primary	Revision	Primary	Revision
Browning et al^4^	3	Retrospective case-control	19	60 (6/54)	60 (6/54)	31.1 ± 10.7	30.9 ± 10.2	Minimum 12	
Cancienne et al^5^	3	Retrospective case-control	21	20 (10/10)	10 (5/5)	29.2 ± 8.6	29.1 ± 8.8	32.9 (24-58)	
Chapman et al^6^	3	Retrospective cohort	22	204 (64/140)	51 (16/35)	33.2 ± 11.2	32.6 ± 10.2	63.9 ± 9.2	
Domb et al^9^	2	Prospective cohort	18	824 (345/479)	97 (31/66)	36.6	33.7	25.7	26.4
Larson et al^17^	3	Retrospective cohort	22	220 (114/106)	79 (35/44)	31.4	29.5	23	26
Maldonado et al^19^	3	Prospective cohort	22	254 (75/179)	127 (33/94)	33.9 ± 13.2	34.9 ± 12.4	72.8 ± 21.2	72.8 ± 23.3
Maldonado et al^18^	3	Retrospective case-control	19	87 (28/59)	87 (29/58)	50.3 ± 7	49.5 ± 8	66.1 ± 36.8	63.5 ± 38.6
Mygind-Klavsen et al^25^	3	Retrospective cohort	18	4154 (1944/2210)	331 (123/208)	38.2 ± 12.7	38 ± 10.6	Minimum 12	
Newman et al^26^	2	Prospective cohort	20	492 (202/290)	246 (101/145)	33.4 ± 9	32.1 ± 9	44 ± 16 (24-97)	43 ± 15 (24-90)
Vogel et al^38^	3	Retrospective case-control	22	72 (20/52)	36 (12/24)	30.5 ± 11.2	31.5 ± 10.3	74.4 ± 21.6	76.8 ± 22.8
Yuro et al^40^	3	Retrospective case-control	20	50 (18/32)	27 (8/19)	47.5 ± 10.5	39.1 ± 8.8	Minimum 24	

a Age and follow-up are reported as mean, mean ± SD, mean ± SD (range), or mean (range) unless otherwise indicated. F, female; LOE, level of evidence; M, male; MINORS, Methodological Index for Non-Randomized Studies.

**Table 2:** Preoperative Osteoarthritis and Intraoperative Labrum and Cartilage Findings^*a*^

Author	Measure	Primary (% of Patients)	Revision (% of Patients)	*P* Value
Browning et al^4^	Tönnis grade	0: 93.3	0: 96.7	NR
Chapman et al^6^	Tönnis grade	1: 6.3	1: 9.5	.503
Domb et al^9^	ALAD	0: 14.8	0: 19.6	.19
		1: 24.3	1: 26.2	.67
		2: 32.3	2: 29.9	.61
		3: 21.7	3: 15.9	.16
		4: 6.4	4: 7.5	.68
	Acetabular Outerbridge	0: 7.3	0: 14	**.02**
		1: 29.8	1: 28	.71
		2: 30.2	2: 29.9	.95
		3: 16.1	3: 15	.76
		4: 10.7	4: 9.3	.66
	Femoral head Outerbridge	0: 68.1	0: 68.2	.98
		1: 1.9	1: 2.8	.81
		2: 6	2: 12.1	**.02**
		3: 6.3	3: 1.9	.06
		4: 5.8	4: 7.5	.49
	Tönnis grade	0: 66.3	0: 66.4	.40
		1: 14.1	1: 17.8	.40
Maldonado et al^19^	Seldes	0: 1.2	0: 9.4	**<.0001**
		1: 37	1: 14.2	
		2: 35.8	2: 53.5	
		1 and 2: 26	1 and 2: 22.8	
	ALAD	0: 13.8	0: 27.6	**.021**
		1: 38.6	1: 30.7	
		2: 24	2: 23.6	
		3: 18.5	3: 13.4	
		4: 5.1	4: 4.7	
	Acetabular Outerbridge	0: 10.6	0: 25.2	**.002**
		1: 40.9	1: 31.5	
		2: 21.7	2: 23.6	
		3: 16.5	3: 9.4	
		4: 10.2	4: 10.2	
	Femoral head Outerbridge	0: 79.1	0: 78.7	.436
		1: 0.8	1: 1.6	
		2: 5.9	2: 9.4	
		3: 8.7	3: 4.7	
		4: 5.5	4: 5.5	
Maldonado et al^18^	Seldes	0: 2.3	0: 20.7	**<.001**
		1: 24.1	1: 11.5	
		2: 32.2	2: 42.5	
		1 and 2: 41.4	1 and 2: 25.3	
	ALAD	0: 23	0: 24.1	.683
		1: 29.9	1: 28.7	
		2: 29.9	2: 24.1	
		3: 9.2	3: 17.2	
		4: 8	4: 5.7	
	Acetabular Outerbridge	0: 23	0: 24.1	.822
		1: 29.9	1: 28.7	
		2: 29.9	2: 24.1	
		3: 9.2	3: 10.3	
		4: 8	4: 12.6	
	Femoral head Outerbridge	0: 77	0: 75.9	.875
		1: 1.1	1: 0	
		2: 11.5	2: 11.5	
		3: 4.6	3: 8	
		4: 5.7	4: 4.6	
	Tönnis grade	0: 79.3	0: 78.2	.853
		1: 20.7	1: 21.8	
Mygind-Klavsen et al^25^	Femoral head ICRS	0-1: 88	0-1: 73	**<.01**
		2-4: 12	2-4: 27	
	Acetabular Beck	0-1: 14	0-1: 33	**<.01**
		2-4: 86	2-4: 67	
Newman et al^26^	≤2-mm joint space	4	5	.706
Vogel et al^38^	Acetabular Beck	3-4: 18.1	3-4: 25	.451
	Femoral head Beck	3-4: 4.2	3-4: 0	.549
	Tönnis grade	0: 90	0: 85	.528
		1: 10	1: 15	
Yuro et al^40^	Labral characteristics	Bruising: 84	Bruising: 70	.160
		Advanced degeneration: 56	Advanced degeneration: 59.3%	.783
	Labral width	<3 mm: 30	<3 mm: 37	.529
		3-8 mm: 58	3-8 mm: 63	.672
		>8 mm: 12	>8 mm: 0	.061
	Acetabular articular cartilage damage	48	33.3	.215
	Tönnis grade	0: 100	0: 92.6	NR
		1: 0	1: 7.4	

a ALAD, acetabular labrum articular disruption; ICRS, International Cartilage Repair Society; NR, not reported. The bolded values indicate significant differences.

### Preoperative and Postoperative Radiographic Parameters

Preoperatively, the LCEA was significantly lower in the primary group (*P* = .03) and alpha angle was significantly higher in the primary group (*P* < .001). The preoperative ACEA and Tönnis angle were not significantly different between the primary and revision groups. Postoperatively, the LCEA, ACEA, alpha angle, and Tönnis angle were not significantly different between the primary and revision groups (Table 3).

Table 3 presents the preoperative and postoperative radiographic parameters in the primary versus revision groups.

**Table 3:** Preoperative and Postoperative Radiographic Parameters^*a*^

	No. of Studies	No. of Patients		Weighted Mean ± SD, deg		MD (95% CI)	*P* Value
		Primary	Revision	Primary	Revision		
Preoperative							
LCEA	9	6197	1162	31.0 ± 6.0	31.1 ± 6.7	0.46 (0.04 to 0.88)	.03
ACEA	6	1347	434	31.3 ± 8.2	30.3 ± 7.5	1.04 (−0.12 to 2.21)	.08
Alpha angle (Dunn)	8	2043	831	62.3 ± 13.2	61.1 ± 24.4	4.69 (2.42 to 6.96)	<.001
Tönnis angle	8	5705	816	5.5 ± 4.5	5.6 ± 4.7	0.13 (−0.40 to 0.67)	.63
Postoperative							
LCEA	5	1392	362	28.5 ± 5.9	29.3 ± 5.8	−0.06 (−0.97 to 0.86)	.91
ACEA	4	1188	311	30.1 ± 6.2	30.4 ± 7.1	0.49 (−0.66 to 1.65)	.40
Alpha angle (Dunn)	6	1464	398	44.8 ± 12.1	44.2 ± 10.9	0.78 (−0.70 to 2.26)	.30
Tönnis angle	5	1392	362	5.4 ± 4.6	5.0 ± 4.9	0.50 (−0.05 to 1.06)	.08

a ACEA, anterior center-edge angle; LCEA, lateral center-edge angle; MD, mean difference.

### Patient-Reported Outcomes

All studies included at least one PRO measure. Overall, 9 of the 11 studies reported whether there were significant differences in PROs between the primary versus revision groups. Of these studies, 7 reported that all postoperative PROs were significantly higher in patients undergoing primary hip arthroscopic surgery. One study reported that while 4 postoperative PROs were significantly higher in the primary group compared with the revision group, 2 PROs were not significantly different between the groups.^6^ Another study reported that in patients undergoing primary versus revision hip labral reconstruction, there were no significant differences in all 5 postoperative PROs between the groups.^38^ Table 4 presents the postoperative PROs reported in each study.

**Table 4:** Patient-Reported Outcomes^*a*^

Author	Measure	Score			MCID			PASS		
		Primary	Revision	*P* Value	Primary	Revision	*P* Value	Primary	Revision	*P* Value
Browning et al^4^	HOS-ADL	86.1 ± 14.4	74.3 ± 22.5	NR	81.7%	53.3%	NR	73.3%	33.3%	NR
	HOS-SSS	76.1 ± 23.8	54.4 ± 33.4	NR	80%	47.4%	NR	67.8%	27.1%	NR
	mHHS	81.7 ± 16.1	72.3 ± 19.9	NR	76.7%	66.7%	NR	75%	33.3%	NR
	iHOT-12	68.5 ± 26.2	57.7 ± 30.9	NR	75%	64.5%	NR	52.6%	29.8%	NR
	VAS pain	2.2 ± 2.3	3.5 ± 2.5	NR	NR					
Cancienne et al^5^	HOS-ADL	80.3 ± 26.7	83.6 ± 11.1	NR	68.8%	90%	NR	55.6%	30%	NR
	HOS-SSS	66.3 ± 34.1	60.5 ± 27.8	NR	78.6%	80%	NR	61.1%	20%	NR
	mHHS	76 ± 25	71.3 ± 12.7	NR	66.7%	80%	NR	66.7%	30%	NR
	VAS pain	2.5 ± 3.6	2.9 ± 1.5	NR	NR					
	VAS satisfaction	7.1 ± 3.9	7.1 ± 1.8	NR	NR					
Chapman et al^6^	HOS-ADL	85.9 ± 15.7	80.4 ± 21	.091	NR		>.275	54%	42%	>.05
	HOS-SSS	75.6 ± 25.3	64.9 ± 32.5	**.034**	NR		>.275	55.8%	38.3%	**.016**
	mHHS	81.2 ± 17.3	72.2 ± 22.4	**.021**	NR		>.275	54%	43%	>.05
	iHOT-12	71.9 ± 25.9	61.4 ± 29.3	**.029**	NR		>.275	60.3%	41.9%	**.001**
	VAS pain	2.6 ± 2.5	3.1 ± 2.7	.213	NR					
	VAS satisfaction	7.8 ± 2.9	6.3 ± 3.5	**.001**	NR					
Domb et al^9^	HOS-ADL	82.3	73.2	**<.005**	NR					
	HOS-SSS	65.5	54.4	**<.005**						
	mHHS	79.3	71	**<.005**						
	VAS pain	2.9	3.8	**<.005**						
	NAHS	80	71.4	**<.005**						
Larson et al^17^	mHHS	88.5	79.9	**<.01**	NR					
	VAS pain	1.7	3	**<.01**						
	SF-12	85	80.1	**<.01**						
Maldonado et al^19^	HOS-SSS	72.7 ± 26.4	59.5 ± 28.9	**.0001**	NR					
	mHHS	84.4 ± 16.7	77.5 ± 19.2	**.001**						
	iHOT-12	73.9 ± 23.8	62.8 ± 26.3	**.0001**						
	VAS pain	2.3 ± 2.3	3.4 ± 2.6	**.0001**						
	NAHS	84 ± 17.1	76 ± 18.6	**<.0001**						
	VAS satisfaction	8.2 ± 2.2	7.5 ± 2.5	**.021**						
Maldonado et al^18^	HOS-SSS	69.6 ± 34.3	54.4 ± 28.7	**.002**	71.2%	66.2%	.53	57.6%	29.4%	**.001**
	mHHS	82.7 ± 18.6	74 ± 19.6	**.006**	72.3%	72.4%	.991	NR		
	iHOT-12	73.7 ± 27.2	59.4 ± 26.6	**<.001**	NR					
	VAS pain	2.4 ± 2.8	3.3 ± 2.4	**.005**	71.1%	82.9%	.078	NR		
	NAHS	81.8 ± 18.7	73.7 ± 17.9	**.002**	76.9%	79.4%	.679	59.8%	31.6%	**<.001**
	VAS satisfaction	8.2 ± 2.6	7.5 ± 2.5	**.017**	NR					
Mygind-Klavsen et al^25^	HAGOS Pain	55 (35-80)	30 (20-50)	**<.01**	NR					
	HAGOS Symptoms	71 (57-86)	54 (43-68)	**<.01**						
	HAGOS ADL	85 (60-95)	60 (40-80)	**<.01**						
	HAGOS Sport/Rec	69 (41-88)	34 (19-61)	**<.01**						
	HAGOS PA	50 (13-75)	13 (0-38)	**<.01**						
	HAGOS QOL	55 (35-80)	30 (20-50)	**<.01**						
	HSAS	3 (2-4)	2 (1-3)	**<.01**						
	NRS pain at rest	9 (1-23)	24 (9-48)	**<.01**						
	NRS pain during activity	13 (2-37)	35 (13-66)	**<.01**						
	EQ-5D	0.78 (0.72-1)	0.72 (0.66-0.78)	**<.01**						
Newman et al^26^	HOS-ADL	87.2 ± 16	79.1 ± 20	**<.001**	NR					
	HOS-SSS	77.1 ± 26	62 ± 30	**<.001**						
	mHHS	82.7 ± 16	79 ± 17	**.039**						
	WOMAC	10.8 ± 13	18 ± 17	**<.001**						
	SF-12 PCS	51.6 ± 9	47 ± 10	**<.001**						
	Tegner	5 (1-10)	4 (2-10)	**.003**						
	VAS satisfaction	9 (1-10)	8 (2-10)	**.024**						
Vogel et al^38^	HOS-ADL	83.5 ± 18.7	81.3 ± 17	.601	80%	70%	.524	65%	48%	.155
	HOS-SSS	71.7 ± 28.4	64.2 ± 29.5	.291	75.7%	68.4%	.751	63%	52%	.461
	mHHS	74.9 ± 19	70 ± 19.5	.295	73.7%	52.9%	.213	65.4%	64%	>.99
	iHOT-12	69.5 ± 26.8	62.6 ± 27.5	.309	78.1%	66.7%	.481	58.5%	45.8%	.332
	VAS pain	25.6 ± 26.7	38.4 ± 32.8	.086	75.6%	53.3%	.118	79.4%	57.7%	.064
Yuro et al^40^	iHOT-12	74.8 ± 27	60.1 ± 29.2	**.03**	92%	66.7%	**.024**	68%	40.7%	**.021**

a Scores are reported as mean, mean ± SD, or median (interquartile range). ADL, Activities of Daily Living; EQ-5D, EuroQol–5 Dimensions; HAGOS, Copenhagen Hip and Groin Outcome Score; HOS-ADL, Hip Outcome Score–Activities of Daily Living; HOS-SSS, Hip Outcome Score–Sports-Specific Subscale; HSAS, Hip Sports Activity Scale; iHOT-12, International Hip Outcome Tool–12; MCID, minimal clinically important difference; mHHS, modified Harris Hip Score; NAHS, Non-Arthritic Hip Score; NR, not reported; NRS, Numeric Rating Scale; PA, Physical Activity; PASS, Patient Acceptable Symptom State; PCS, physical component summary; QOL, Quality of Life; SF-12, 12-Item Short Form Health Survey; Sport/Rec, Sports and Recreation; VAS, visual analog scale; WOMAC, Western Ontario and McMaster Universities Osteoarthritis Index.

#### Meta-analysis of Patient-Reported Outcomes

The HOS-ADL score was reported in 5 studies, with 848 patients versus 503 patients undergoing primary versus revision hip arthroscopic surgery, respectively. The weighted mean HOS-ADL score was 86.3 ± 16.4 versus 78.9 ± 20.1 for the primary versus revision groups, respectively. The HOS-ADL score was significantly higher in patients undergoing primary hip arthroscopic surgery (MD, 6.59 [95% CI, 3.10-10.07]; *I*^2^ = 41%; *P* = .0002).

The HOS-SSS score was reported in 7 studies, with 1189 patients versus 717 patients undergoing primary versus revision hip arthroscopic surgery, respectively. The weighted mean HOS-SSS score was 74.8 ± 26.9 versus 60.3 ± 30.1 for the primary versus revision groups, respectively. The HOS-SSS score was significantly higher in patients undergoing primary hip arthroscopic surgery (MD, 14.26 [95% CI, 11.53-16.99]; *I*^2^ = 0%; *P* < .00001).

The mHHS score was reported in 7 studies, with 1189 patients versus 717 patients undergoing primary versus revision hip arthroscopic surgery, respectively. The weighted mean mHHS score was 82.4 ± 17.1 versus 76.5 ± 18.6 for the primary versus revision groups, respectively. The mHHS score was significantly higher in patients undergoing primary hip arthroscopic surgery (MD, 5.82 [95% CI, 3.92-7.72]; *I*^2^ = 9%; *P* < .00001).

The VAS pain score was reported in 6 studies, with 697 patients versus 371 patients undergoing primary versus revision hip arthroscopic surgery, respectively. The weighted mean VAS pain score was 2.4 ± 2.5 versus 3.4 ± 2.6 for the primary versus revision groups, respectively. The VAS pain score was significantly lower in patients undergoing primary hip arthroscopic surgery (MD, −0.98 [95% CI, −1.31 to −0.64]; *I*^2^ = 0%; *P* < .00001).

The iHOT-12 score was reported in 6 studies, with 727 patients versus 388 patients undergoing primary versus revision hip arthroscopic surgery, respectively. The weighted mean iHOT-12 score was 72.5 ± 25.5 versus 60.9 ± 27.7 for the primary versus revision groups, respectively. The iHOT-12 score was significantly higher in patients undergoing primary hip arthroscopic surgery (MD, 11.38 [95% CI, 7.98-14.79]; *I*^2^ = 0%; *P* < .00001).

The NAHS score was reported in 2 studies, with 341 patients versus 214 patients undergoing primary versus revision hip arthroscopic surgery, respectively. The weighted mean NAHS score was 83.4 ± 17.5 versus 75.1 ± 18.3 for the primary versus revision groups, respectively. The NAHS score was significantly higher in patients undergoing primary hip arthroscopic surgery (MD, 8.03 [95% CI, 4.89-11.18]; *I*^2^ = 0%; *P* < .00001).

**Figure 2.**
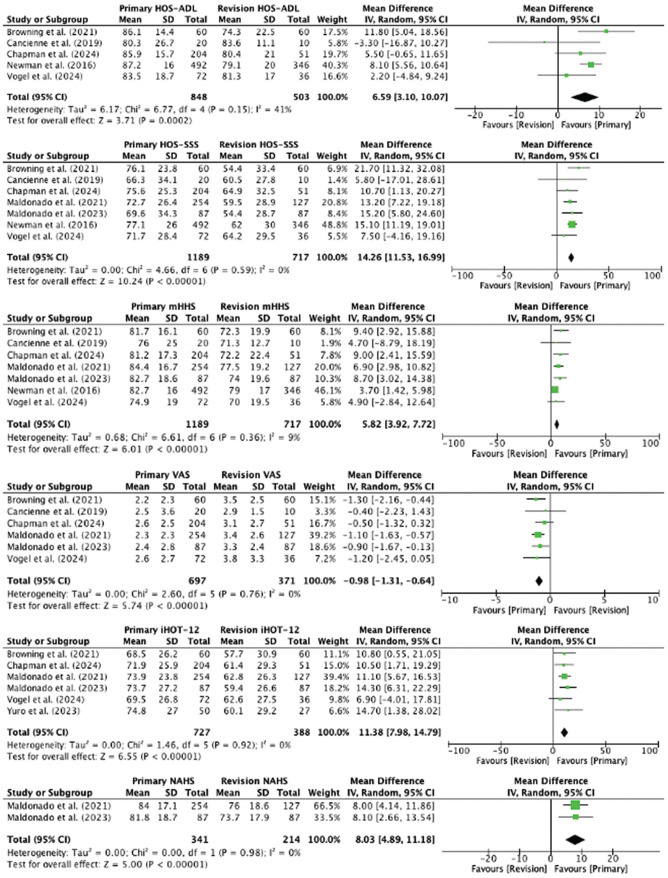
Forest plots comparing the mean difference in postoperative patient-reported outcomes between primary and revision hip arthroscopic surgery.

Figure 2 contains forest plots comparing the MD for the HOS-ADL, HOS-SSS, mHHS, VAS pain, iHOT-12, and NAHS scores between the primary and revision groups.

### Clinically Significant Outcomes

The achievement rate of the minimal clinically important difference (MCID) for at least one PRO measure was reported in 5 studies. The rate of achieving the MCID for any PRO measure for the primary versus revision groups ranged from 66.7% to 92% versus 47.4% to 90%, respectively. The rates of achieving the MCID for primary versus revision hip arthroscopic surgery were as follows for specific PRO measures: HOS-ADL (68.8%-81.7% vs 53.3%-90%, respectively), HOS-SSS (71.2%-80% vs 47.4%-80%, respectively), mHHS (66.7%-76.7% vs 52.9%-80%, respectively), iHOT-12 (75%-92% vs 64.5%-66.7%, respectively), and VAS pain (71.1%-75.6% vs 53.3%-82.9%, respectively). A total of 4 studies reported whether there were significant differences in MCID achievement rates for at least one PRO measure between the primary and revision groups. Of the 4 studies, 1 found a significantly higher MCID achievement rate for at least one PRO measure in the primary group.

The achievement rate of the Patient Acceptable Symptom State (PASS) for at least one PRO measure was reported in 6 studies. The rate of achieving the PASS for any PRO measure for the primary versus revision groups ranged from 52.6% to 79.4% versus 20% to 64%, respectively. The rates of achieving the PASS for primary versus revision hip arthroscopic surgery were as follows for specific PRO measures: HOS-ADL (54%-73.3% vs 30%-48%, respectively), HOS-SSS (55.8%-67.8% vs 20%-52%, respectively), mHHS (54%-75% vs 30%-64%, respectively), and iHOT-12 (52.6%-68% vs 29.8%-45.8%, respectively). A total of 4 studies reported whether there were significant differences in PASS achievement rates for at least one PRO measure between the primary and revision groups. Of the 4 studies, 3 found a significantly higher PASS achievement rate for at least one PRO measure in the primary group. Achievement rates of the MCID and PASS are reported in Table 4.

### Adverse Outcomes

#### Complications

Surgical complications were reported in 4 studies, with 1150 patients versus 211 patients undergoing primary versus revision hip arthroscopic surgery, respectively. One study reported the overall complication rate between primary versus revision groups, but did not differentiate between specific complications.^9^ The pooled complication rate was 4.9% and 7.4% for the primary and revision groups, respectively. The risk of complications was significantly higher in patients undergoing revision hip arthroscopic surgery (RR, 0.55 [95% CI, 0.31-0.97]; *I*^2^ = 0%; *P* = .04). Table 5 details the complications reported by these studies.

**Table 5:** Complications^*a*^

Author	Primary	Revision	*P* Value
Chapman et al^6^	Neuropathy (0.5%)	Neuropathy (3.9%)	.102
Domb et al^9^	Neurapraxia of LFCN (0.3%), neurapraxia of pudendal nerve (0.4%), neurapraxia of sciatic nerve (0.6%), CRPS in distribution of saphenous nerve (0.1%), heterotopic ossification (1.3%), superficial wound infection that resolved with oral antibiotics (0.5%), DVT (0.5%), pulmonary embolism (0.2%), deep surgical site infection (0.1%), stress fracture (0.3%), ACS (0.1%)		.34
Vogel et al^38^	Transient neuropathy of pudendal nerve (1.4%), superficial wound infection that resolved with oral antibiotics (1.4%)	Transient neuropathy of pudendal nerve (2.7%)	>.99
Yuro et al^40^	None	None	>.99

a ACS, abdominal compartment syndrome; CRPS, complex regional pain syndrome; DVT, deep vein thrombosis; LFCN, lateral femoral cutaneous nerve.

#### Revision Surgery

The incidence of revision surgery was reported in 5 studies, with 1647 patients versus 462 patients undergoing primary versus revision hip arthroscopic surgery, respectively. The pooled revision rate was 8.1% versus 5.6% for the primary versus revision groups, respectively (RR, 1.07 [95% CI, 0.49-2.36]; *I*^2^ = 63%; *P* = .86).

#### Conversion to THA

The incidence of conversion to THA was reported in 6 studies, with 1735 patients versus 550 patients undergoing primary versus revision hip arthroscopic surgery, respectively. The pooled rate of conversion to THA was 5.1% versus 10.0% for the primary versus revision groups, respectively. The risk of conversion to THA was significantly higher in patients undergoing revision hip arthroscopic surgery (RR, 0.51 [95% CI, 0.36-0.73]; *I*^2^ = 0%; *P* = .0002). Overall, 3 studies^9,18,19^ reported that the relative risk of conversion to THA was significantly higher in patients undergoing revision hip arthroscopic surgery (range of relative risk, 2.0-2.63), whereas 1 study^17^ reported that the relative risk of conversion to THA was significantly higher in patients undergoing primary hip arthroscopic surgery (relative risk, 3.9).

**Figure 3.**
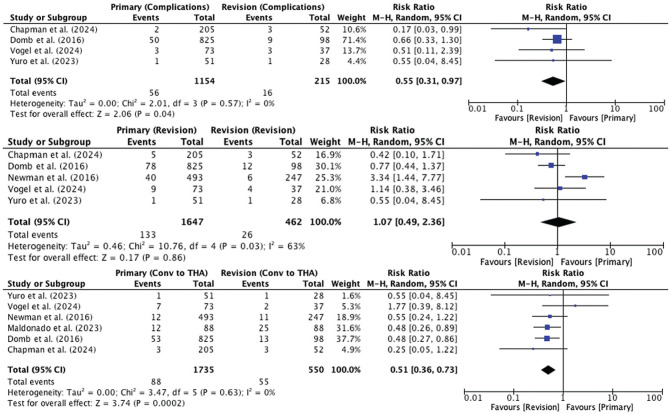
Forest plots comparing the risk ratio for adverse outcomes in patients undergoing primary versus revision hip arthroscopic surgery.

Figure 3 contains forest plots comparing the risk of complications, revision, and conversion to THA between the primary and revision groups.

## Discussion

In this systematic review and meta-analysis of 11 studies that compared the outcomes of primary versus revision hip arthroscopic surgery, the most important findings were that patients undergoing revision hip arthroscopic surgery had significantly lower postoperative HOS-ADL, HOS-SSS, mHHS, iHOT-12, and NAHS scores; significantly higher postoperative VAS pain scores; and a significantly higher risk of complications and conversion to THA. Radiographic parameters for the pooled groups were clinically similar between patients undergoing primary versus revision hip arthroscopic surgery. Despite similar postoperative correction of the alpha angle for a cam deformity in both the primary and revision groups, the percentage of patients who achieved clinically significant improvements in PROs was generally higher in the primary setting compared with the revision setting.

The primary versus revision groups in this systematic review consisted of 56.1% versus 65.3% female patients, respectively. Female sex has been associated with greater rates of dysplasia, instability, and generalized joint hyperlaxity, with a recent systematic review reporting that 14 of 48 included studies cited female sex to be a negative predictor of postoperative outcomes.^21^ In a study consisting of 595 patients (61.7% female) undergoing primary hip arthroscopic surgery, multivariate regression analysis using revision hip arthroscopic surgery as an endpoint found that female sex was significantly associated with the need for revision (rate ratio, 2.86 [95% CI, 1.43-5.73]; *P* = .002).^11^ In another study comparing differences between successful versus failed (mHHS score <80 or subsequent hip surgery) primary hip arthroscopic surgery for acetabular retroversion, the authors found that female patients who underwent femoral osteoplasty (*P* < .05) or both femoral and acetabular osteoplasty (*P* < .03) had significantly higher failure rates compared with their male counterparts.^31^ Furthermore, in 2 large database studies evaluating the outcomes of primary hip arthroscopic surgery, female sex was significantly associated with revision hip arthroscopic surgery (odds ratio, 1.6 [95% CI, 1.1-2.3]; *P* < .001)^16^ and conversion to THA (odds ratio, 1.68 [95% CI, 1.41-2.01]; *P* < .001).^20^

While both preoperative and postoperative radiographic parameters were not clinically significant between the groups, none of the studies in this analysis explored the association between radiographic parameters and the success or failure of hip arthroscopic surgery. In contrast, many studies in the literature highlighted specific radiographic predictors of clinical failure after hip arthroscopic procedures. A recent study comparing successful versus failed revision hip arthroscopic surgery found that the failed group demonstrated a significantly smaller decrease in the alpha angle preoperatively to postoperatively. Specifically, each 1° increase in the difference between the preoperative and postoperative alpha angle was associated with a 17% decrease in the odds of failure.^1^ Additionally, a systematic review of 14 studies by de Sa et al^34^ demonstrated that correction of the alpha angle in cam-type FAI to a minimum of 55° was associated with improved outcomes, corresponding with the long-held notion that inadequate resection of cam-type FAI morphology remains a leading cause of failure of primary hip arthroscopic surgery. A recent study found that larger preoperative alpha angles were associated with earlier achievement of the MCID and maximal outcome improvement satisfaction threshold for the mHHS and NAHS.^29^ In the present systematic review, the mean alpha angle ranged from 61.1° to 62.3° preoperatively to 44.2° to 44.8° postoperatively, while the mean LCEA, ACEA, and Tönnis angle remained essentially unchanged postoperatively in both groups, which can explain why the PROs improved in both groups. McQuivey et al^22^ reported that in patients undergoing primary hip arthroscopic surgery, higher preoperative Tönnis angles, indicating a more dysplastic hip, were significantly associated with secondary surgery. The odds ratio for secondary surgery was 1.12 (95% CI, 1.0-1.2; *P* = .05) per 1° increase in the preoperative Tönnis angle. Furthermore, a systematic review evaluating risk factors associated with failed primary hip arthroscopic surgery found that a preoperative LCEA >33° was significantly associated with the need for revision hip arthroscopic surgery.^36^ Therefore, a careful evaluation of preoperative and postoperative radiographic parameters, in addition to the magnitude of their change, may be beneficial to adequately manage patients’ expectations and potential outcomes after hip arthroscopic surgery.^12,24^

Larson et al^17^ evaluated significant predictive variables associated with better outcomes after revision hip arthroscopic surgery, as indicated by the mHHS score. They found that the identification and treatment of subspine/anterior inferior iliac spine impingement (*P* = .014), an increased postoperative femoral offset (*P* = .024), labral repair/reconstruction (*P* = .009), and capsular repair/plication (*P* = .032) were associated with significantly better mHHS scores. However, sex, age, time from initial surgery, FAI morphology, alpha angle, femoral neck-shaft angle, LCEA, Tönnis angle, grade 4 chondral lesion, psoas tenotomy, and ligamentum teres tears were not associated with significantly better mHHS scores. This emphasizes the importance of the management of subspine/anterior inferior iliac spine impingement, labral preservation/reconstruction, the restoration of capsular integrity, and the treatment of cam-type FAI with regard to offset in all hip arthroscopic surgery cases, primary or revision.

The use of the MCID and PASS for changes in PROs allows for the establishment of psychometric benchmarks that represent (1) whether a clinically meaningful change is present and (2) whether patients actually feel that their condition has improved.^2,27^ A noticeable finding in this study was that the rates of achieving the PASS across all PRO measures for the primary and revision groups ranged from 52.6% to 79.4% versus 20% to 64%, respectively, with no overlap in ranges for the HOS-ADL, HOS-SSS, and iHOT-12. This suggests that despite proper correction of radiographic parameters during revision hip arthroscopic surgery, PROs may still suffer. This corresponds with the widely believed notion that patient outcomes are optimized if surgical treatment is performed well in the primary setting, whereas there may be a ceiling effect for outcomes after revision surgery caused by irreversible structural, biological, and psychological effects from having undergone previous surgery.^15,23,28^ Across 4 studies with a total of 12 PRO measures reporting on the significance of PASS achievement rates, 5 PRO measures had significantly higher PASS achievement rates in the primary group, and the remaining 7 PRO measures had no significant differences between the primary and revision groups. For both the MCID and PASS, achievement rates were either significantly higher in the primary group or not significantly different between the primary and revision groups.

### Limitations

The results of this study should be considered in the context of their limitations. Hip arthroscopic surgery is used to address a wide array of hip abnormalities, such as FAI, labral tears, and chondral lesions. The arthroscopic procedures performed in the studies analyzed in this systematic review were mixed, as presented in Table 2, which may have influenced the outcomes. However, the patient undergoing revision hip arthroscopic surgery represents a unique case that may often require atypical approaches compared with a primary case, a factor that is difficult to control for. Yuro et al^40^ was the only study in which primary and revision cases underwent the same procedure: labral reconstruction. Interestingly, they reported no significant differences in PROs, MCID and PASS achievement rates, complications, revision rates, and rates of conversion to THA. Additionally, while 8 studies matched patients based on characteristics and comorbidities, 3 studies did not.^9,25,40^ While this may introduce a degree of heterogeneity, the results of our meta-analysis demonstrated mostly low with some moderate heterogeneity, as indicated by the *I*^2^ values ranging from 0% to 67%. Furthermore, this systematic review was biased toward the single study by Mygind-Klavsen et al,^25^ which contributed 4154 of the 6437 patients undergoing primary hip arthroscopic surgery. However, patients from Mygind-Klavsen et al’s^25^ study were not included in the meta-analysis, given that the study reported unique PROs along with no report of adverse events between the primary and revision groups. While there were nearly equal percentages of male and female patients undergoing primary surgery, nearly two-thirds of patients undergoing revision surgery were female. As discussed earlier, female sex has been associated with less favorable surgical outcomes in the literature, which may explain why more female patients underwent revision surgery and why revision surgery outcomes may have been inferior to primary surgery outcomes. Finally, while all 11 studies reported PROs, only 6 individual studies reported on whether patients experienced clinically significant outcomes as indicated by the MCID or PASS.

## Conclusion

In this systematic review and meta-analysis of studies that compared the outcomes of primary versus revision hip arthroscopic surgery, patients undergoing revision hip arthroscopic surgery were less likely to achieve clinically significant improvements in postoperative PROs and exhibited a higher risk of complications and conversion to THA compared with patients undergoing primary hip arthroscopic surgery. These findings suggest that outcomes are optimized in the primary setting, and surgeons should appropriately counsel patients regarding expectations after revision hip arthroscopic surgery.

## Supplemental Material

sj-pdf-1-ajs-10.1177_03635465251324944 – Supplemental material for Clinical Outcomes of Primary Versus Revision Hip Arthroscopic Surgery: A Systematic Review and Meta-analysis Supplemental material, sj-pdf-1-ajs-10.1177_03635465251324944 for Clinical Outcomes of Primary Versus Revision Hip Arthroscopic Surgery: A Systematic Review and Meta-analysis by Muzammil Akhtar, Daniel Razick, Mustafa Jundi, Jamal Zahir, Sonia Aamer, Anand Dhaliwal, Trevor Shelton and Dean Wang in The American Journal of Sports Medicine
