# Supplementary material for: Clinical Outcomes of Primary Versus Revision Hip Arthroscopic Surgery: A Systematic Review and Meta-analysis
Source: Am J Sports Med. 2025 Mar 30;53(12):3025–36. doi: 10.1177/03635465251324944 (PMC12489170; doi:10.1177/03635465251324944)
Supplement: sj-pdf-1-ajs-10.1177_03635465251324944 – Supplemental material for Clinical Outcomes of Primary Versus Revision Hip Arthroscopic Surgery: A Systematic Review and Meta-analysis [file sj-pdf-1-ajs-10.1177_03635465251324944.pdf]

Clinical Outcomes of Primary Versus Revision Hip Arthroscopy: A Systematic Review and Meta-analysis

Appendix Table A1: Indications for Surgery

Indications for Surgery		
**Author**	**Primary**	**Revision**
Browning et al. [4]	FAI	Residual FAI, adhesions, capsular defect or insufficiency, cartilage damage, labral tear or insufficiency, or foreign body removal
Cancienne et al. [5]	FAI in setting of BHD (LCEA of 18° to 25°)	Continued hip pain, labral retear, or residual cam lesion
Chapman et al. [6]	FAI and labral tears	Residual cam lesion, labral retear, capsular defect or insufficiency, or heterotopic ossification
Domb et al. [9]	Predominantly labral tears	Predominantly labral tears and residual FAI
Larson et al. [17]	FAI	Residual cam and/or pincer FAI (alpha angle >50°, head-neck offset <8 mm, focal acetabular retroversion with anterior/lateral/posterior overcoverage, and LCEA >35°)
Maldonado et al. (2021) [19]	FAI, chondral damage, greater trochanteric pain syndrome, internal snapping, acetabular osteophytes	Not specified
Maldonado et al. (2023) [18]	FAI	Residual FAI and labral tears
Mygind-Klavsen et al. [25]	FAI	Adhesions or scar tissue inside joint, insufficient healing of labrum, insufficient acetabuloplasty and femoral head-neck osteochondroplasty, internal snapping hip syndrome, new labral or cartilage injury, heterotopic ossification, intraarticular loose body, defect in hip capsule, or rupture of ligamentum teres
Newman et al. [26]	FAI	Not specified
Vogel et al. [38]	FAI in setting of BHD (LCEA of 18° to 25°)	Anterior impingement, residual cam FAI, labral retear, adhesions, or capsular insufficiency
Yuro et al. [40]	Labral tears	Labral retear or capsular insufficiency

FAI, Femoroacetabular impingement; LCEA, lateral center-edge angle; BHD, borderline hip dysplasia

Appendix Table A2: Procedures Performed

Author	Procedures Performed		
	Primary	Revision	Significant Differences
Browning et al.	Labral debridement (100%), labral repair (100%), labral reconstruction (0%), acetabuloplasty (100%), femoral osteochondroplasty (100%), capsular closure/plication (100%), capsular reconstruction (0%), removal of foreign body (0%), synovectomy (100%)	Labral debridement (88.3%), labral repair (83.3%), labral reconstruction (5%), acetabular rim trimming (81.7%), femoral osteochondroplasty (86.7%), capsular closure/plication (76.7%), capsular reconstruction (23.3%), removal of foreign body (20%), synovectomy (80%)	Not reported
Cancienne et al.	All patients in each cohort underwent diagnostic hip arthroscopy, arthroscopic labral repair, synovectomy, femoral osteochondroplasty, and capsular plication		No significant difference
Chapman et al.	Labral debridement (99%), labral repair (98%), labral reconstruction (0%), acetabuloplasty (92.6%), femoral osteochondroplasty (98.5%), capsular plication (98%), capsular reconstruction (0%)	Labral debridement (96.1%), labral repair (82.4%), labral reconstruction (5.9%), acetabuloplasty (72.9%), femoral osteochondroplasty (95.8%), capsular plication (92.2%), capsular reconstruction (23.5%)	Significantly greater incidence of labral repair and acetabuloplasty in primary group. Significantly greater incidence of labral reconstruction and capsular reconstruction in revision group.
Domb et al.	Labral debridement (35.6%), labral repair (59.3%), labral reconstruction (1.8%), acetabuloplasty (71.5%), acetabular microfracture (9.6%), acetabular subchondral cyst removal (3.5%), femoroplasty (69.2%), femoral head microfracture (1.3%), femoral head chondroplasty (8.8%), femoral head subchondral cyst removal (3.5%), capsular repair (44.9%), capsular release (50.2%), partial capsulotomy (1.9%), removal of loose bodies (14.1%), removal of heterotopic ossification (0.2%)	Labral debridement (57%), labral repair (25.2%), labral reconstruction (8.4%), acetabuloplasty (48.6%), acetabular microfracture (10.3%), acetabular subchondral cyst removal (1.9%), femoroplasty (55.1%), femoral head microfracture (1.2%), femoral head chondroplasty (6.5%), femoral head subchondral cyst removal (1.9%), capsular repair (32.7%), capsular release (57%), partial capsulotomy (0.9%), removal of loose bodies (24.3%), removal of heterotopic ossification (6.5%)	Significantly greater incidence of acetabuloplasty, femoroplasty, labral repair, and capsular repair in primary group. Significantly greater incidence of removal of loose bodies, removal of heterotopic ossification, labral debridement, and labral reconstruction in revision group.
Larson et al.	Not reported	Labral debridement (31.8%), labral repair (57.6%), labral reconstruction (8.2%), capsular plication for prior unrepaired capsulotomy and/or capsular hypermobility/hyperlaxity (27%), microfracture for full thickness chondral defect (8.2%), removal of adhesions (63.5%)	Not reported
Maldonado et al. (2021)	Labral debridement (31.9%), labral repair (64.6%), labral reconstruction (3.1%), acetabuloplasty (68.1%), acetabular microfracture (6.7%), acetabular chondroplasty (15.4%), acetabular subchondral cyst removal (5.9%), femoroplasty (79.5%), femoral head microfracture (1.6%), femoral head chondroplasty (6.3%), femoral head subchondral cyst removal (5.9%), capsular repair (55.1%), interportal capsulotomy without closure (44.9%)	Labral debridement (59.8%), labral repair (23.6%), labral reconstruction (7.1%), acetabuloplasty (44.9%), acetabular microfracture (9.4%), acetabular chondroplasty (25.2%), acetabular subchondral cyst removal (1.6%), femoroplasty (59.8%), femoral head microfracture (3.1%), femoral head chondroplasty (4.7%), femoral head subchondral cyst removal (1.6%), capsular repair (39.4%), interportal capsulotomy without closure (60.6%)	Significantly greater incidence of acetabuloplasty, femoroplasty, labral repair, and capsular repair in primary group. Significantly greater incidence of acetabular chondroplasty and labral debridement in revision group.
Maldonado et al. (2023)	Labral debridement (34.5%), labral repair (58.6%), labral reconstruction (4.6%), acetabuloplasty (65.5%), acetabular microfracture (6.9%), femoroplasty (88.5%), femoral head microfracture (1.1%), interportal capsulotomy without repair (50.6%), capsular repair (49.5%)	Labral debridement (51.7%), labral repair (20.7%), labral reconstruction (9.2%), acetabuloplasty (41.4%), acetabular microfracture (8%), femoroplasty (57.5%), femoral head microfracture (2.3%), interportal capsulotomy without repair (75.9%), capsular repair (24.1%)	Significantly greater incidence of labral repair, capsular repair, femoroplasty, and acetabuloplasty in primary group. Significantly greater incidence of labral debridement and interportal capsulotomy without repair in revision group.
Mygind-Klavsen et al.	Labral debridement (17%), labral repair (83%), labral reconstruction (0%), acetabuloplasty (81%), acetabular microfracture (7%), femoral head-neck osteochondroplasty (89%), femoral head microfracture (0%)	Labral debridement (37%), labral repair (61%), labral reconstruction (2%), acetabuloplasty (58%), acetabular microfracture (5%), femoral head-neck osteochondroplasty (86%), femoral head microfracture (2%)	Significantly greater incidence of acetabuloplasty and labral repair in primary group. Significantly greater incidence of labral debridement in revision group.
Newman et al.	Labral debridement (2%), labral repair (92%), labral reconstruction (7%), acetabuloplasty (4%), acetabular microfracture (8%), femoroplasty (9%),	Labral debridement (13%), labral repair (27%), labral reconstruction (48%), acetabuloplasty (6%), acetabular microfracture (9%), femoroplasty (20%), femoral head	Significantly greater incidence of labral repair, femoral head microfracture, and acetabuloplasty + femoroplasty

	femoral head microfracture (3%), acetabuloplasty + femoroplasty (88%)	microfracture (11%), acetabuloplasty + femoroplasty (61%)	in primary group. Significantly greater incidence of labral debridement, labral reconstruction, femoroplasty, and femoral head microfracture in revision group.
Vogel et al.	Labral debridement (13.9%), labral repair (86.1%), acetabuloplasty (86.1%), femoroplasty (100%), microfracture (2.8%), capsular repair (100%)	Labral debridement (22.2%), labral repair (77.8%), acetabuloplasty (77.8%), femoroplasty (77.8%), microfracture (2.8%), capsular repair (100%)	Significantly greater incidence of femoroplasty in primary group.
Yuro et al.	Labral reconstruction (100%), acetabuloplasty (50%), acetabular chondroplasty (54%), femoroplasty (84%)	Labral reconstruction (100%), acetabuloplasty (38%), acetabular chondroplasty (50%), femoroplasty (92%)	Not reported
